# Five and four dimensional experiments for robust backbone resonance assignment of large intrinsically disordered proteins: application to Tau3x protein

**DOI:** 10.1007/s10858-016-0048-7

**Published:** 2016-07-18

**Authors:** Szymon Żerko, Piotr Byrski, Paweł Włodarczyk-Pruszyński, Michał Górka, Karin Ledolter, Eliezer Masliah, Robert Konrat, Wiktor Koźmiński

**Affiliations:** 1 Faculty of Chemistry, Biological and Chemical Research Centre, University of Warsaw, 02093 Warsaw, Poland; 2 Section of Biophysics, Faculty of Physics, University of Warsaw, 02093 Warsaw, Poland; 3 Department of Computational and Structural Biology, Max F. Perutz Laboratories, University of Vienna, Vienna, Austria; 4 Departments of Neuroscience and Pathology, University of California, San Diego, La Jolla, CA 92093 USA

**Keywords:** Intrinsically disordered proteins, Resonance assignment, High-dimensionality NMR, Non-uniform sampling, Isotropic mixing, MOCCA-XY16

## Abstract

New experiments dedicated for large IDPs backbone resonance assignment are presented. The most distinctive feature of all described techniques is the employment of MOCCA-XY16 mixing sequences to obtain effective magnetization transfers between carbonyl carbon backbone nuclei. The proposed 4 and 5 dimensional experiments provide a high dispersion of obtained signals making them suitable for use in the case of large IDPs (application to 354 a. a. residues of Tau protein 3x isoform is presented) as well as provide both forward and backward connectivities. What is more, connecting short chains interrupted with proline residues is also possible. All the experiments employ non-uniform sampling.

**Electronic supplementary material:**

The online version of this article (doi:10.1007/s10858-016-0048-7) contains supplementary material, which is available to authorized users.

## Introduction

Intrinsically disordered proteins (IDPs) or protein regions (IDPRs) have found general interest in the recent molecular biology research (Wright and Dyson 1999; Habchi et al. 2014). These proteins are not only lacking stably folded tertiary structures but also their intrinsic flexibility has significant impact on their biological functionality, therefore challenging the old structure-function paradigm.

Nuclear magnetic resonance spectroscopy (NMR) is nowadays one of the most efficient spectroscopic techniques in the life sciences, providing insight into molecular structure and dynamics. In recent years, the fast progress of NMR methodology and applications can be observed with special attention paid to difficult, but biologically relevant systems such as IDPs. In contrast to well-folded globular proteins, the peculiar properties of IDPs introduce additional challenges that need to be overcome to obtain spectra enabling effective resonance assignment. The conformational dynamics exhibited by IDPs lead to severe averaging of chemical shifts, which are mostly determined by the amino acid chemical composition and the protein’s primary structure. At the same time, however, this dynamic behavior leads to favorable relaxation properties, and allows high dimensionality and long evolution periods within pulse sequences. Prolines are often abundant in IDPs, thus further complicating the resonance assignment procedure.

Application of sparse non-uniform sampling (NUS) in NMR experiments enabled resolution enhancement and high-dimensionality without prohibitively long experiment durations, by the acquisition of only a small fraction of data points required conventionally (Mobli and Hoch 2008; Coggins et al. 2010; Orekhov and Jaravine 2011; Freeman and Kupče 2012; Hiller and Wider 2012; Kazimierczuk et al. 2012). In the case of an experiment producing a small number of spectral peaks featuring a limited range of amplitudes, reasonably good spectra can be obtained by employing simple zero-augmented Fourier transformation. However, when artefacts due to incomplete sampling are comparable or stronger than weaker peaks, the spectrum should be reconstructed in order to remove artefacts.

Till now, several strategies were proposed for the reconstruction of sparsely sampled data sets. Among them: maximum entropy (Robin et al. 1991), multidimensional decomposition (MDD) (Orekhov and Jaravine 2011), compressed sensing (CS) (Kazimierczuk and Orekhov 2011; Holland et al. 2011), and signal separation algorithm (SSA) (Stanek and Koźmiński 2010; Stanek et al. 2012). All of them proved their usability for different kinds of experiments.

The approaches for effective resonance assignment of IDPs include automated projection spectroscopy (APSY) (Hiller et al. 2005; Narayanan et al. 2010; Yao et al. 2014), HA (Mäntylahti et al. 2010; Yao et al. 2014) and direct ^13^C detection (Bermel et al. 2006, 2009; Nováček et al. 2011; Pantoja-Uceda and Santoro 2013), as well as high-dimensional (4–7D) non-uniformly sampled experiments (Kazimierczuk et al. 2013; Nowakowski et al. 2015). In contrast to well folded proteins, where the use of aliphatic ^1^H and ^13^C chemical shifts proved their usefulness, in IDPs the aliphatic chemical shifts depend mostly on the residue type. In the case of IDP molecules, the most resolved are the backbone CO and N chemical shifts, thus leading to the “CON–CON” approach (Piai et al. 2014; Żerko and Koźmiński 2015).

The aim of present study was to design new techniques capable of providing robust sequential resonance assignment of disordered proteins not only of moderate size but also for larger constructs, as illustrated by a full CO, N, N^H^ resonance assignment of 354 a. a. Tau protein 3x isoform. To achieve these goals two 5 dimensional: (H)NCOCONH and (HACA)CON(CO)CONH techniques were designed and compared. The use of MOCCA-XY16 block for performing CO–CO tocsy mixing as a crucial magnetization transfer between neighbouring amino acid residues was chosen. Both experiments provide robust connectivities through CO_(i−1)_N_(i)_ chemical shift pairs. 3D HNCO was used as the base spectrum in the Sparse Multidimensional Fourier Transform (Kazimierczuk et al. 2009) processing routine. High dimensionality secures good signal separation while NUS allows to achieve high resolution in indirectly detected dimensions. The first experiment, 5D (H)NCOCONH, is a direct expansion of a 3 dimensional experiment presented by Yoshimura et al. (2015) who already proved its capability of performing assignment of non-proline resonances of α-synuclein. Moreover, he showed that obtaining cross-peaks from *i* ± 3 residues is possible when long enough mixing time is used. Nevertheless, in our case, it was decided to attune mixing time to provide *i* ± 1 connectivities as lower number of peaks present in the spectra simplify data analysis as well as leads to a lower sampling noise level. The second experiment, 5D (HACA)CON(CO)CONH, exploits Hα excitation. It improves S/N ratio in the case of effectively exchangeable H^N^ residues, but more importantly, makes the resonance assignment of proline residues fairly easy. Taking into account the presence of both *i* ± 1 cross-peaks, it allows reliable resonance assignment of proline abundant protein sequence fragments, which is often the case in IDPs. In comparison to other 5D approaches a significant advantage is the correlation not only with antecedent (*i* − 1) but also with a following (*i* + 1) residue. Aforementioned approach presented by Yoshimura et al. (2015) used only 3D spectra basing on peak separation in the ^1^H,^15^N dimensions. In the figure S1 the comparison of 3D and 4D spectra, acquired for Tau3x protein is provided. It clearly shows significantly lower level of peak overlap owing to additional CO dimension in 4D spectra, which facilitates unambiguous spectra analysis. Another important parameter is the B_0_ field strength used. Higher field brings improvements in both sensitivity and resolution, on the other hand, this effect is counterbalanced by an increase in relaxation loses during the CO evolution and mixing periods. In fact, MOCCA-XY16 mixing sequence minimizes relaxation loses (Furrer et al. 2004) since the magnetization is transverse only during 180° pulses. Nevertheless, such relaxation loses become significant for long mixing periods. Since relaxation of CO nuclei is dominated by the chemical shift anisotropy mechanism, the effectiveness of which scales up with the square of B_0_, the use of higher B_0_ fields may not be an optimal choice. Hence, proposed pulse sequences’ performance was tested on 600 and 800 MHz spectrometers.

In principle, the resonance assignment strategy based on a 3D HNCO and a single 5D experiment (HACA)CON(CO)CONH could provide complete (including proline residues) and robust data for a backbone (H^N^, N and CO) resonance assignment. The application to Tau3x isoform proved that this ideal case in not as far from reality as one could imagine. First, application of both 5D experiments to a sample of α-synuclein, a medium size (140 a.a.) IDP is presented. Finally, application of two complementary 4D experiments derived from 5D (HACA)CON(CO)CONH performed on a Tau3x protein sample is discussed and compared with results obtained using a different set of 5D experiments.

## Materials and methods

### Pulse sequences

**Fig. 1:**
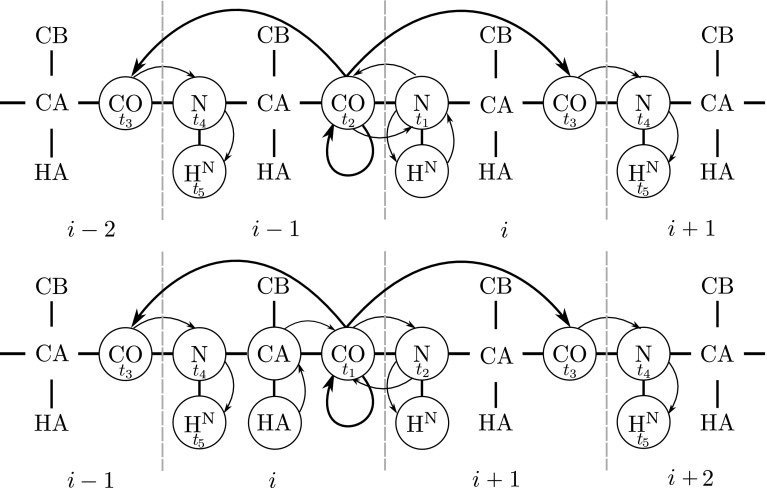
Coherence pathway schemes for 5D (H)NCOCONH (*top*) and 5D (HACA)CON(CO)CONH (*bottom*) experiments

**Fig. 2:**
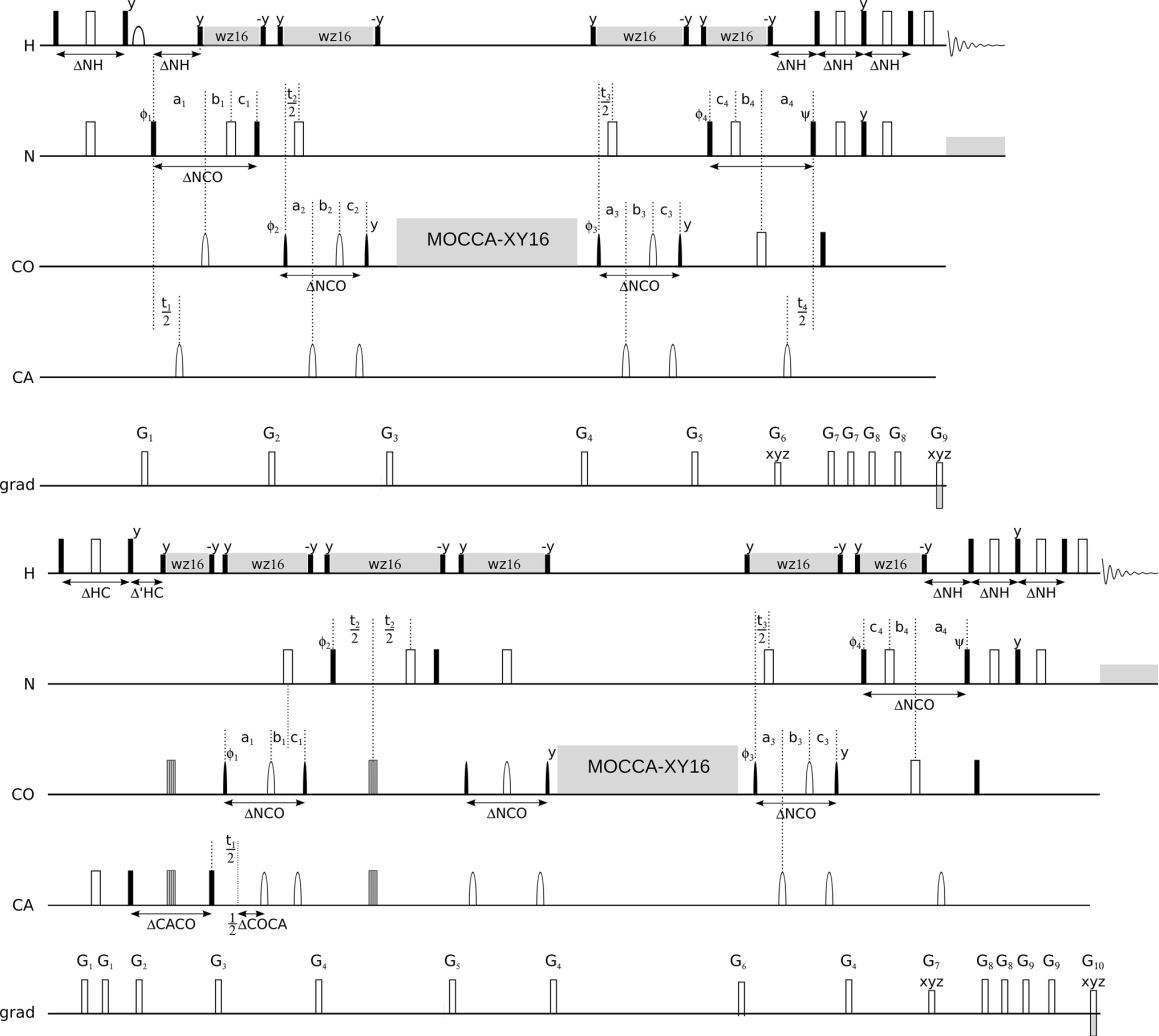
(H)NCOCONH (*top*) and (HACA)CON(CO)CONH (*bottom*) experiments pulse sequence schemes. *Rectangles* represent hard pulses. *Filled* and *empty symbols* represent 90° and 180° pulses, respectively. ^1^H and ^15^N composite pulse decoupling is performed with WALTZ-16 (Shaka et al. 1983), at γB_1_/2π of 5.1 and 1.14 kHz at 800 MHz spectrometer, and 3.37 and 0.97 kHz at 600 MHz, respectively. Simultaneous inversion of CA and CO spins is achieved using a 6-element composite pulse (Shaka 1985). Selective CA and CO ^13^C pulses are applied with the RF field strength adjusted to |ΔΩ_CA − CO_|/√15 (√3) for 90° and 180° pulses, respectively. For the 800 MHz spectrometer 90° and 180°, rectangular and sinc-shaped pulsed (*bell-shaped* at the scheme) with a duration of 40.1 (35.9) µs, and 65.8 (58.8) µs, respectively, are used. Whereas, for the 600 MHz spectrometer the respective pulse lengths are 53.5 (47.9) µs and 87.8 (78.4) µs. Off-resonance pulses are applied using phase modulation of the carrier. The PFG durations are set to 0.5 ms, except for coherence selection gradients for which 2.0 and 0.2 ms, are used. Delay durations are set as follows: ΔNH = 5.4 ms, ΔNCO = 28 ms, ΔCOCA = 9.1 ms. Evolution periods for H, N, CO are in semi-constant-time mode: a_i_ = (t_i_ + Δ)/2; b_i_ = t_i_(1 − Δ/t_imax_)/2; c_i_ = Δ(1 − t_i_/t_imax_)/2 or in constant-time mode: a_i_ = (t_i_ + Δ)/2; b_i_ = 0; c_i_ = (Δ − t_i_)/2 where Δ stands for ΔNH, ΔNCO. In the second sequence evolution for N in t_2_ is in real-time mode. The four step phase cycle is: ϕ_1_ = x, −x; ϕ_2_ = 2x, 2(−x) and ϕ_rec_ = x, 2(−x), x. In t_1_, t_2_, t_3_, t_4_ dimensions quadrature is accomplished using States-TPPI method, by incrementing ϕ_1_, ϕ_2_, ϕ_3_, ϕ_4_ phases, respectively. The phase ψ = x is inverted simultaneously with the last gradient pulse to achieve echo-antiecho coherence transfer selection in the last indirect dimension. The coherence selection gradients marked with *xyz* are applied at the magic angle (600 MHz) or along the z axis (800 MHz). 180° water 1.42 (1.07) ms sinc-shaped flipback pulses are used, on the 600 and 800 MHz spectrometers, respectively

Magnetization transfers schemes and pulse sequences of 5D (H)NCOCONH and 5D (HACA)CON(CO)CONH are provided in the Figs. 1 and 2. The radiofrequency (RF) field of the CO-selective 180° pulses in MOCCA-XY16 was set at 4.68 (6.23) kHz with a duration of the 180° pulse (*d*) of 107.0 (80.2) μs on the 600 (800) MHz spectrometer, which was determined in order to ensure that Cα nuclei experience an effective 720° rotation during each carbonyl 180° pulse (Felli et al. 2009). It is possible to choose pulse power to get 360° rotation for Cα but it would significantly increase the amount of energy dissipated at the RF coil. Furthermore, 720° rotation choice leads to narrower inversion profiles but in the case of IDPs such narrow bandwidths are still sufficient to cover all involved nuclei. Value of the 180° pulse repetition period Δ (Δ is calculated including 180° pulse length) was initially set to 500 μs. Mixing time was attuned to provide magnetizations transfers between consecutive CO nuclei securing the presence of *i* ± 1 cross-peaks.

### NMR spectroscopy

All measurements were performed on Agilent DD2 600 and 800 MHz spectrometers equipped with standard triple resonance room temperature probes. In all experiments: four scans were acquired for each FID, relaxation delay of 1.3 s was used, mixing time was set to 250 ms and temperature to 288 K. Δ of 500 and 250 μs was used for α-synuclein and Tau3x protein measurements, respectively. Detailed spectral widths and maximal evolution times are given in the Supporting Information Table S1. The pulse sequence codes for Varian/Agilent spectrometers are available from the authors on demand.

For 5D spectra 3D HNCO was used as an input for Sparse Multidimensional Fourier Transform procedure (Kazimierczuk et al. 2009). All spectra were processed using ToASTD (Kazimierczuk et al. 2006), additionally cleaner3d (Stanek and Koźmiński 2010), cleaner4d (Stanek et al. 2012), and reduced (Kazimierczuk et al. 2009) programmes were used for processing 3D, 4D and 5D spectra, respectively. All used processing software is available at http://nmr.cent3.uw.edu.pl/software. All spectra were inspected using the Sparky program (Goddard and Kneller 2000), Nmrglue Python package was used for visualization purposes (Helmus and Jaroniec 2013). The whole spectra analysis and resonance assignment were performed manually.

### Sample preparation

NMR sample of α-synuclein contained 1 mM of ^13^C,^15^N-labeled α-synuclein in a 20 mM sodium phosphate buffer, pH 6.5, 200 mM NaCl, 10 % D_2_O.

NMR sample of Tau3x contained 0.5 mM of ^13^C,^15^N-labeled Tau3x in 20 mM NAP, 1 mM β-Merc.EtOH, 1 mM EDTA, 1 mM NaN_3_, 100 mM NaCl, 10 % D_2_O.

## Results and discussion

### Application to α-synuclein

**Fig. 3:**
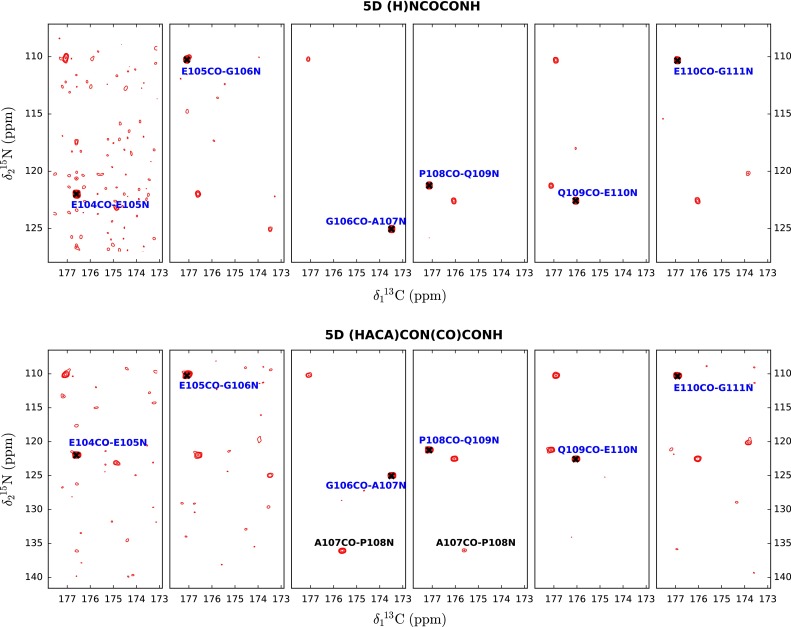
Resulting 2D cross-sections from 5D (H)NCOCONH (*top*) and 5D (HACA)CON(C)CONH for residues 105–111 of α-synuclein (strip plot for P108 is not shown). Diagonal peak positions are marked with *black crosses*. Note the presence of cross-peaks A107CO-P108N on 3rd and 4th panels (*bottom*), which allow sequential assignment despite the presence of a proline residue

A crucial acquisition parameter is the length of the mixing sequence. To find an efficient duration of the mixing time two (H)NCOCONH spectra were recorded with mixing times set to 150 and 250 ms, respectively. It appears that for the employed MOCCA-XY16 implementation longer mixing times are preferred (see columns 1 and 2 in the Table 1) and 250 ms mixing time was used for all further acquisitions. Since, the rate of coherence transfer does not depend on the field strength, the value established at 800 MHz, due to better relaxation properties at lower field, is always a safe choice. The next step of the study was to compare the performance of the two proposed pulse sequences: (H)NCOCONH and (HACA)CON(CO)CONH (see Fig. 3 for the examples of obtained 2D cross-sections). Note that, since S/N ratio in the case of non-uniformly sampled spectra is an ill-defined parameter (Hyberts et al. 2013), to objectively evaluate the results from different acquisition setups the number of identified peaks was compared. As one can see from comparison of the results presented in the columns 2 and 3, Table 1. (HACA)CON(CO)CONH technique provides slightly more complete results. Note, that this experiment additionally allows effective resonance assignment of proline residues. The smaller influence of the H^N^ exchange on the performance of 5D (HACA)CON(CO)CONH seems to counterbalance the use of a longer and more complicated pulse sequence.

**Table 1:** Comparison of results obtained with different parameters for 5D (H)NCOCONH and 5D (HACA)CON(CO)CONH experiments for the α-synuclein sample

	1	2	3
Backwards links	25 % (32/128)	77 % (99/128)	79 % (105/133)
Forward links	22 % (28/128)	71 % (91/128)	79 % (105/133)
Number of points	1100	1100	1100
Mixing time (ms)	150	250	250
Spectrometer (MHz)	800	800	800
Pulse sequence	(H)NCOCONH	(H)NCOCONH	(HACA)CON(CO)CONH

To finally evaluate the performance of the proposed (HACA)CON(CO)CONH experiment, two longer (71 h long each) acquisitions were performed on two different spectrometers, 600 and 800 MHz (see Table 2). Although the results of the measurements on both spectrometers were comparable, surprisingly, the lower (600 MHz) field spectrometer provided slightly more information.

**Table 2:** Comparison of results obtained from 5D (HACA)CON(CO)CONH experiment using 600 and 800 MHz spectrometers for the α-synuclein sample

	1	2
Backwards links	90 % (120/133)	92 % (123/133)
Forward links	88 % (117/133)	94 % (125/133)
Number of points	2250	2250
Mixing time (ms)	250	250
Spectrometer (MHz)	800	600
Pulse sequence	(HACA)CON(CO)CONH	(HACA)CON(CO)CONH

### Application to Tau3x isoform

**Fig. 4:**
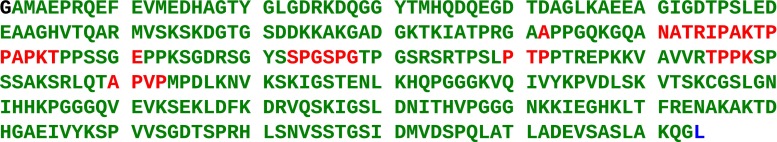
Tau3x protein sequence with resonance assignment of CON pairs obtained using: 3D HNCO, 5D HN(CA)CONH, 5D (HACA)CON(CA)CONH, 5Dn HabCabCONH and 5D HC(CC-TOCSY)CONH. *Green* CON pair assigned, *black* only CO assigned, *blue* only N assigned, *red* no assignment

The resonance assignment of Tau3x isoform was first attempted using a set of experiments: 3D HNCO, 5D HN(CA)CONH (Kazimierczuk et al. 2010) and 5D (HACA)CON(CA)CONH (Zawadzka-Kazimierczuk et al. 2012). Additionally, for easy amino acid residue type determination 5D HabCabCONH (Kazimierczuk et al. 2010) and 5D HC(CC-TOCSY)CONH (Kazimierczuk et al. 2009; Hiller et al. 2008) were recorded. All spectra were recorded using a 800 MHz spectrometer equipped with a standard triple resonance room-temperature probe, total measurement time for all 5D experiments was 92 h. The obtained results are depicted in Fig. 4. As one can see, the vast majority of the residues have been assigned (328 out of 353 CO_i−1_N_i_ pairs) which would ordinarily be considered a satisfactory result. Nevertheless, all of the unassigned CO_i−1_N_i_ pairs are located in proline rich fragments of Tau3x protein sequence, clearly showing that the assignment procedure was limited by insufficient information gained from analysed spectra. In other words, for a full assignment of such regions a different approach is needed.

**Fig. 5:**
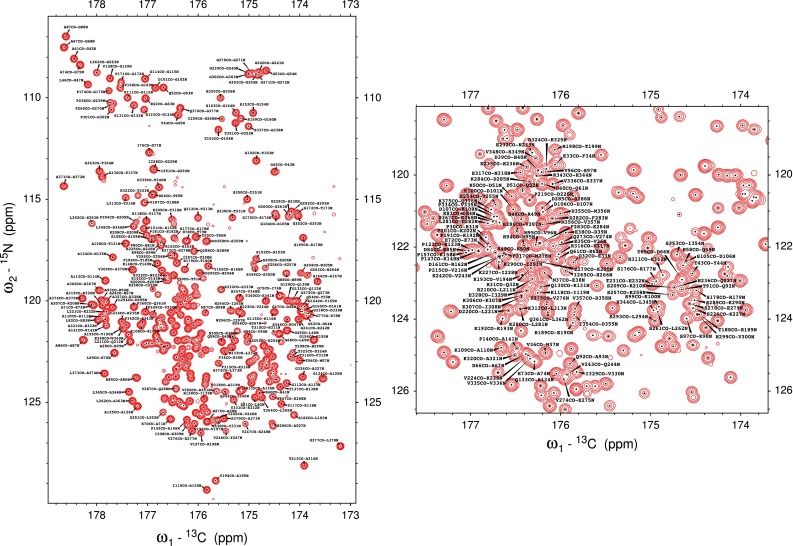
Tau3x CON projection taken from a 3D HNCO spectrum (*left*). Zoom into the most crowded part of the CON projection (*right*)

Knowing that sampling artefacts will be even more troublesome in the case of a larger protein and taking into account the incomplete set of signals obtained for the α-synuclein sample, suggesting limited sensitivity of the proposed techniques, it was decided to lower the dimensionality of experiments used for Tau3x resonance assignment. Application of 4D spectra allows access to a vast number of efficient reconstruction methods, which suppress sampling artefacts (i.e. Maximum entropy, signal separation algorithm). In addition, 4D experiments are more sensitive, on the other hand they provide more ambiguous data. Naturally, different choice of 4 from 5 dimensions in a 4D experiment derived from the proposed 5D experiment is possible. In our approach each 4D experiment provides only one new chemical shift (CO or N) correlating it with the three chemical shifts already known from 3D HNCO—we call this the “1 + 3” approach. Obviously several “2 + 2” 4D experiments could be proposed (i.e. CON + H^N^,N; CON + H^N^,CO;). We concluded that all of these “2 + 2” possibilities are less favourable, mainly because the main bottleneck in the analysis of resultant spectra of all of them would be the fact that none of 2D correlations could provide sufficiently dispersed peaks. Even the most desired CO_(i−1)_N_(i)_ correlation 2D spectrum becomes crowded in the case of relatively big proteins (see Fig. 5) and providing additional H^N^ chemical shift can significantly reduce this problem. Another drawback of a CON + COH^N^ approach would be the lack of sensitivity enhancement that would compromise the signal to noise ratio in such a 4D experiment.

**Fig. 6:**
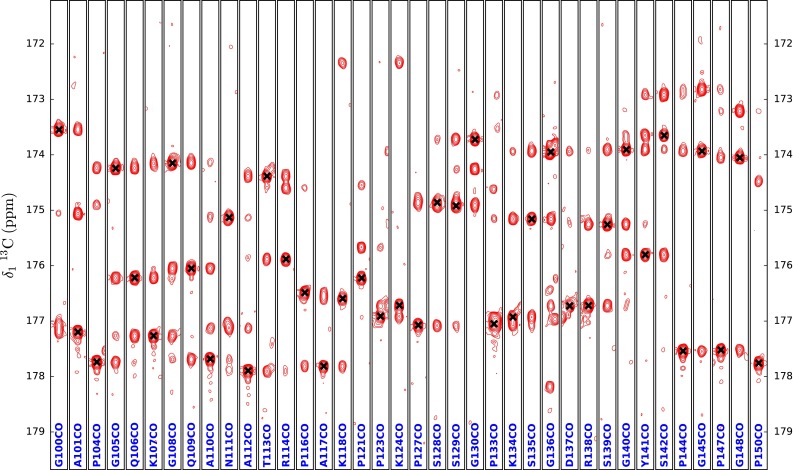
Strip plots for the residues from A101 to G151 taken from 4D (HACA)CO(NCO)CONH of Tau3x. Diagonal peak positions are marked with *black crosses*. Strips plots for proline residues are not shown. ^13^C chemical shifts of residues preceding prolines are evaluated from cross-peaks present on neighbouring residues’ strip plots

**Fig. 7:**
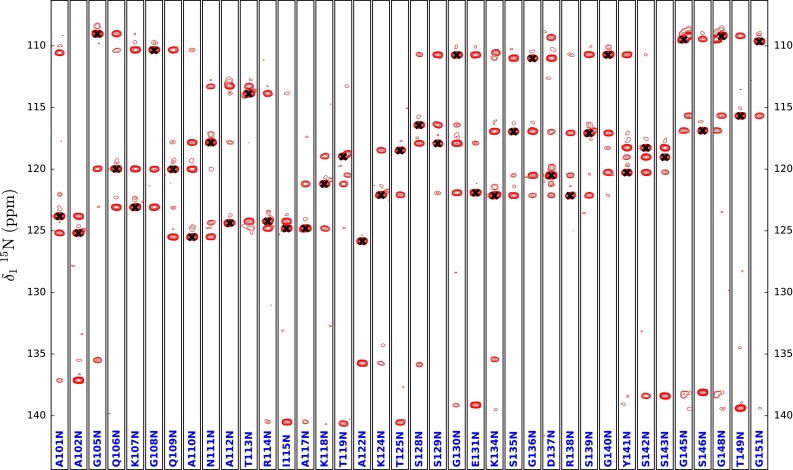
Strip plots for the residues from A101 to G151 taken from 4D (HACACO)N(CO)CONH of Tau3x. Diagonal peak positions are marked with *black crosses*. Strips plots for proline residues are not shown, nonetheless their ^15^N chemical shifts are evaluated from cross-peaks present on neighbouring residues’ strip plots. The small, lower ^15^N frequency peaks originate from ^2^H^15^N–^1^H^15^N isotopomers

The second attempt used only the 3D HNCO, and two complementary 4D spectra: (HACA)CO(NCO)CONH and (HACACO)N(CO)CONH (66 h long each). All spectra were recorded using a 600 MHz spectrometer equipped with a standard triple resonance room-temperature probe. The 4D experiments used correlate CO_(i−1)_ and N(i) chemical shifts with signals present in the 3D HNCO. As a result, 4D spectra can be inspected in a manner similar to the usual 3D triple resonance experiments used for protein sequential resonance assignment (see Figs. 6, 7). The only difference is that instead of ^1^H, ^15^N-HSQC a 3D HNCO is treated as a base for the analysis of strip plots obtained from 4D experiments, which allows for a disambiguation of spectra analysis, as the resonance assignment process of IDPs is hampered by a severe peak overlap (see figure S1 for a comparison of peaks separation provided by the used 4D experiments with 3D (HACACO)N(COCO)NH and 3D (HACA)CO(NCOCO)NH). Both 4D spectra were processed using the cleaner4d program (Stanek et al. 2012) performing spectral reconstruction using the SSA algorithm, currently the only one capable of efficient processing of such high resolution 4D data sets, with file sizes reaching 140 GBs. What is worth mentioning, no additional spectra providing Cα and/or Cβ chemical shifts were used to aid with the identification of residue type, as it was not found to be necessary. Since a 600 MHz spectrometer was used, relaxation loses during tocsy mixing were significantly suppressed in comparison to the 800 MHz spectrometer, and to improve mixing effectiveness taking advantage of lower power load at 600 MHz, it was decided to use a shorter Δ value of 250 µs.

**Fig. 8:**
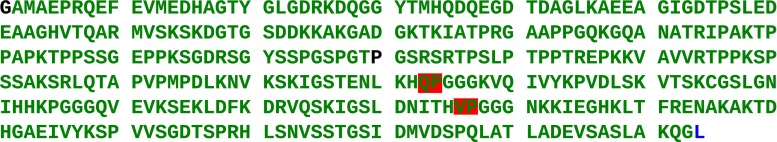
Tau3x protein sequence with resonance assignment of CON pairs obtained using 3D HNCO, 4D (HACA)CO(NCO)CONH and 4D (HACACO)N(CO)CONH. *Green* CON pair assigned, *black* only CO assigned, *blue* only N assigned. *Red* *background* indicates correction of a previous assignment

The final results are depicted in Fig. 8. Only 1G and 150P N and 354L CO chemical shifts are missing. Additionally, the mixed-up assignments of 213–214 and 276–277 residues were identified and corrected. Overall performance of the strategy used is more than satisfactory, as an almost full assignment was obtained, even though the experiments were performed using a standard 600 MHz spectrometer and without the use of an expensive cryogenically cooled probe.

## Discussion

The backbone (H^N^, N and CO) resonance assignment obtained with (4)5D (HACA)CON(CO)CONH could be easily complemented with Cα/β and Hα/β chemical shifts, for example with 5D HabCabCONH. It would be even possible to incorporate evolutions of CA and HA chemical shifts within the presented experiments in a similar manner to the recently presented hybrid of sparse random sampling and projection spectroscopy (Żerko and Koźmiński 2015). Resonance assignment can be further expanded to the side chains using 5D HC(CC-TOCSY)CONH leading to a full signal assignment.

In our study of Tau3x isoform, 5D (HACA)CON(CO)CONH did not provide sufficient sensitivity. It should be possible to overcome this limitation by utilising a cryogenically cooled probe, especially employing shaped salt tolerant NMR tubes. Spectral quality would surely benefit from the removal of sampling artefacts which at present is not possible for 5 dimensional spectra.

Results obtained from a set of two 4D spectra are more than satisfactory. In similar challenging studies of big disordered proteins comparably complete resonance assignment was obtained only for the MAP2c protein (Nováček et al. 2013). Previous studies of proteins from the Tau family provided less complete assignments than this study (Narayanan et al. 2010; Yao et al. 2014). What is more, all of aforementioned studies were performed on significantly more concentrated samples and using far more expensive high-field spectrometers equipped with cryogenically cooled probes, which demonstrates the superiority of the novel approach presented herein.

## Conclusions

We presented new 4D and 5D experiments for the backbone resonance assignment of intrinsically disordered proteins. Described techniques use CO–CO tocsy type magnetization transfers and are especially effective in the resonance assignment of proline rich sequence fragments. We proved that these experiments can be successfully run on a standard room-temperature probe, even on a medium field NMR spectrometer (600 MHz).

## Electronic supplementary material

Below is the link to the electronic supplementary material.

Supplementary material 1 (PDF 1757 kb)
