# Supplementary material for: Five and four dimensional experiments for robust backbone resonance assignment of large intrinsically disordered proteins: application to Tau3x protein
Source: J Biomol NMR. 2016 Jul 18;65:193–203. doi: 10.1007/s10858-016-0048-7 (PMC4983291; doi:10.1007/s10858-016-0048-7)
Supplement: Supplementary file 1 — Supplementary material 1 (PDF 1757 kb) [file 10858_2016_48_MOESM1_ESM.pdf]

Supporting information

for

Five and four dimensional experiments for robust backbone resonance assignment of large intrinsically disordered proteins. Application to Tau 3x protein.

Szymon Żerko[1], Piotr Byrski[1], Paweł Włodarczyk-Pruszyński[1], Michał Górka[1,2], Karin Ledolter[3], Eliezer Masliah[4], Robert Konrat[3], Wiktor Koźmiński[1*]

[1] *Faculty of Chemistry, Biological and Chemical Research Centre, University of Warsaw, Warsaw, 02093, Poland*

[2] *Faculty of Physics, Section of Biophysics, University of Warsaw, Warsaw, 02093, Poland*

[3] *Department of Computational and Structural Biology, Max F. Perutz Laboratories, University of Vienna, Vienna, Austria*

[4] *Departments of Neuroscience and Pathology, University of California, San Diego, La Jolla, CA 92093, USA*

* To whom correspondence should be addressed

kozmin@chem.uw.edu.pl

Table. S1 Experimental parameters of performed NMR experiments.

Experiment	Sample	Spectrometer [MHz]	Spectral width [Hz] & maximal evolution time [ms] for each indirect detected dimension			
3D HNCO	α-synuclein	800			^{13}C 2600 75	^{15}N 3000 75
3D HNCO	α-synuclein	600			^{13}C 1500 100	^{15}N 1800 100
5D (H)NCOCONH	α-synuclein	800	^{15}N 2500 30	^{13}C 3000 30	^{13}C 3000 30	^{15}N 2500 30
5D (HACA)CON(CO)CONH	α-synuclein	800	^{15}N 4000 30	^{13}C 3000 30	^{13}C 3000 30	^{15}N 2500 30
5D (HACA)CON(CO)CONH	α-synuclein	600	^{15}N 3000 30	^{13}C 2250 30	^{13}C 2250 30	^{15}N 1870 30
3D HNCO	Tau3x	800			^{13}C 2700 100	^{15}N 2000 100
3D HNCO	Tau3x	600			^{13}C 1700 125	^{15}N 2000 125
5D HN(CA)CONH	Tau3x	800	^{1}H 1500 20	^{15}N 2000 40	^{13}C 2700 40	^{15}N 2000 40
5D (HACA)CON(CA)CONH	Tau3x	800	^{15}N 4400 40	^{13}C 2700 40	^{13}C 2700 40	^{15}N 2000 40
5D HabCabCONH	Tau3x	800	^{1}H 6400 15	^{13}C 14000 7	^{13}C 2700 40	^{15}N 2000 40
5D 5D HC(CC-TOCSY)CONH	Tau3x	800	^{1}H 4500 15	^{13}C 14000 7	^{13}C 2700 40	^{15}N 2000 40
4D (HACA)CO(NCO)CONH	Tau3x	600		^{13}C 1700 50	^{13}C 1700 50	^{15}N 2000 50
4D (HACACO)N(CO)CONH	Tau3x	600		^{15}N 3000 50	^{13}C 1700 50	^{15}N 2000 50
3D (HACA)CO(NCOCO)NH	Tau3x	600		^{13}C 1700 50		^{15}N 2000 50
3D (HACACO)N(COCO)NH	Tau3x	600		^{15}N 3000 50		^{15}N 2000 50

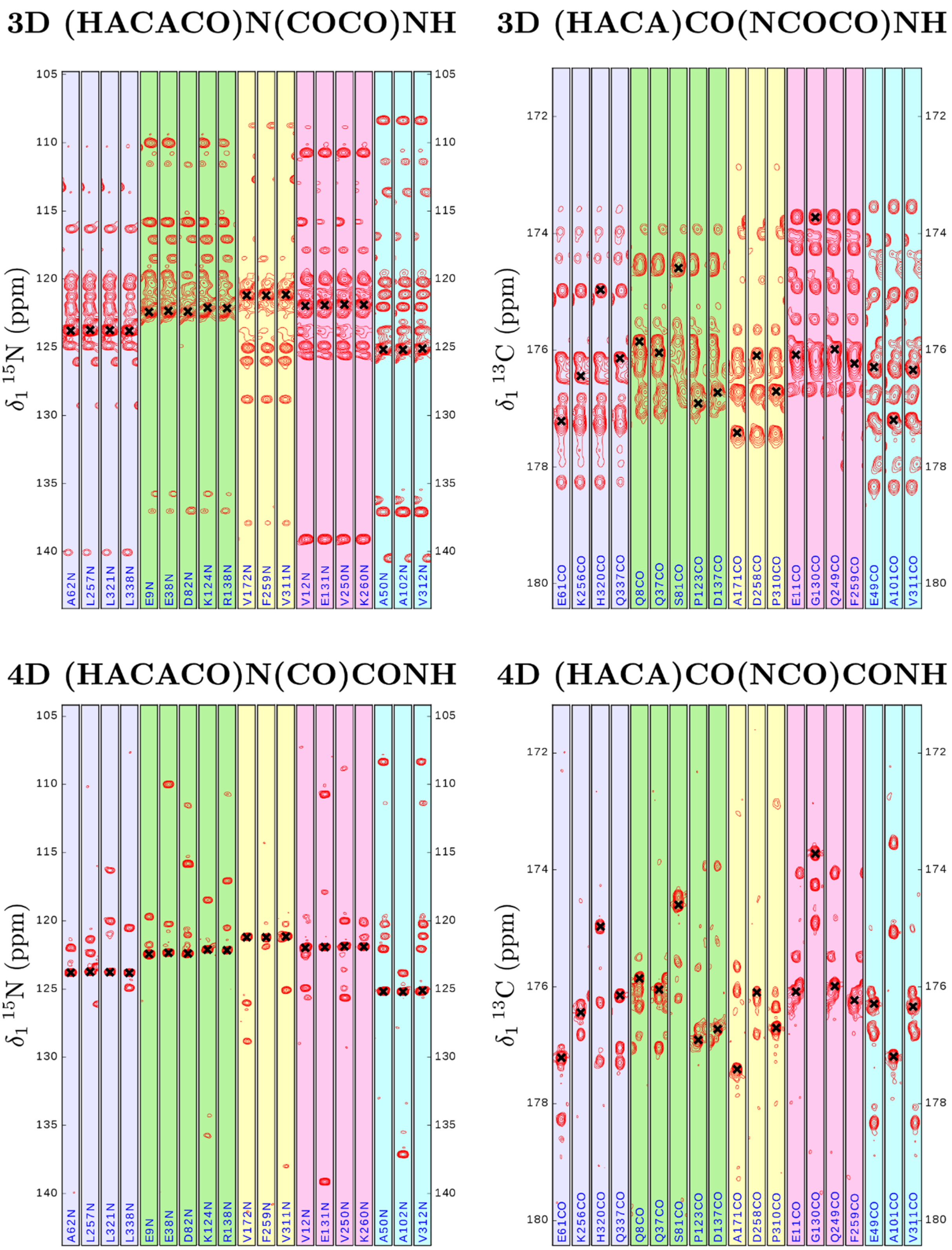

Figure. S1 Comparison of peak separation between 3D and 4D spectra of Tau3x protein acquired on 600 MHz spectrometer in the same time (66 h each). Diagonal peak positions are marked with black crosses. The same background colour is used for peaks overlapping in ^{1}H,^{15}N dimensions.
